# Pravastatin Transdermal Patches: Effect of The Formulation and Two Different Lengths of Microneedles on *In-vitro* Percutaneous Absorption Studies

**DOI:** 10.22037/ijpr.2019.1100914

**Published:** 2020

**Authors:** Pablo Serrano Castañeda, José Juan Escobar-Chávez, Johana Arroyo Vázquez, Isabel Marlen Rodríguez Cruz, Luz María Melgoza Contreras

**Affiliations:** a *Facultad de Estudios Superiores Cuautitlán-Universidad Nacional Autónoma de México. Unidad de Investigación Multidisciplinaria. Laboratorio 12: Sistemas Transdérmicos Carretera Cuautitlán Teoloyucan, km 2.5 San Sebastián Xhala C.P 54714 Cuautitlán Izcalli, Estado de México. *; b *Universidad Autónoma Metropolitana-Departamento de Sistemas Biológicos. UAM-Xochimilco. Calzada del Hueso 1100. Colonia Villa Quietud. C.P. 04960. Ciudad de México. *; c *Hospital Regional de Alta Especialidad de Zumpango. Unidad de Enseñanza e Investigación. Zumpango Edo. de Méx. Carretera Zumpango-Jilotzingo #400. Barrio de Santiago 2da Sección. Zumpango, Estado de México. C.P. 55600.*

**Keywords:** Transdermal patch, Pluronic F-127, Skin, Pravastatin, Solid microneedles

## Abstract

Transdermal patches loaded with pravastatin was previously characterized in another published study by Serrano-Castañeda *et al*; 2015. These transdermal patches (TP) were generated by the plate casting technique, the *in-vitro* percutaneous absorption studies of TP were evaluated for three different formulations with different quantities of Pluronic F-127 (PF-127): i) without PF-127 (TP W), ii) 1% of PF-127 (TP 1%), and iii) 3% of PF-127 (TP 3%) using solid microneedles as a penetration enhancer with two different lengths: i) 0.25 mm and ii) 2.25 mm and iii) *in-vitro* permeation studies by passive diffusion. The fluxes (F), time lag (t_Lag_) and permeability constants (Kp) for each formulation were: TP W (F:38.5µg/cm^2^*h, t_Lag_:18.97h and Kp:5.9x10^-3^ cm/h), TP W with microneedles of 0.25 mm (F:103.3 µg/cm^2^*h, t_Lag_: 20.76 h and Kp: 0.0158 cm/h), TP W and microneedles of 2.25 mm (F:105.2µg/cm^2^*h, t_Lag_: 21.16 h and Kp: 0.0159cm/h), TP 1% (F:90 µg/cm^2^*h, t_Lag_: 19.48 h and Kp: 0.0137 cm/h), TP 1% with microneedles of 0.25 mm (F:111.4µg/cm^2^*h, t_Lag_:19.11h and Kp:0.017cm/h), and TP 1% with microneedles of 2.25 mm (F:115.2µg/cm^2^*h, t_Lag_:16.73h and Kp:0.017cm/h), TP 3% (F:40.9µg/cm^2^*h, t_Lag_:20.45h and Kp:0.0062 cm/h), TP 3% with microneedles of 0.25 mm (F:67.1 µg/cm^2^*h, t_Lag_: 21.79h and Kp:0.0102cm/h) and TP 3% with microneedles of 2.25 (F:70.5 µg/cm^2^*h, t_Lag_:20.44h and Kp:0.0107cm/h). Results show that the formulation of TP affects the pravastatina flux and Kp parameters, however the length of microneedles only has important effect on t_Lag_.

## Introductions

Hypercholesterolemia is a high level of cholesterol in the blood and is related with high risk of atherosclerosis, heart attacks or stroke. It is one of the principal causes of death in the world. The treatment is oral (statins) by forever. The oral route of administration presents some drawbacks such as irritation of the gastrointestinal tract, drug interactions with food, variability associated with the oral route, “first-pass” metabolism and also multiple dosages are required. This may cause some omission and consequently generate therapeutic ineffectiveness (1-4).

The transdermal patch improves the treatment of hypercholesterolemia because they allow the drug to be released into the systemic circulation (3). These systems have a controlled and constant delivery of drugs through the skin. They have a prolonged release, thus avoiding adverse effects to the oral route, and a reduction of variability in absorption. These systems are non-invasive, comfortable for the patient and most of the patients with hypercholesterolemia have multi-therapy which increases drug interaction as well as interaction with food at the gastrointestinal level. When transdermally administered these interactions are avoided. Another advantage is that they avoid the first pass hepatic metabolism (1,2,4).

Pravastatin sodium is a statin that acts as an inhibitor of 3-hydroxy-3-methylglutaryl-coenzyme A reductase (HMG-CoA reductase), which is used to treat hypercholesterolemia. This enzyme catalyzes the conversion of 3-hydroxy-3-methylglutaryl-coenzyme A (HMG-CoA) to mevalonate, which is an early and rate-limiting step in the biosynthesis of cholesterol (5, 6). Pravastatin sodium has good characteristics to be formulated in a transdermal patch as a low molecular weight (424.528 g/mol), the low initial dosage in some cases suffers a high first-pass metabolism and it has an absolute bioavailability of 17%. These characteristics make pravastatin sodium an interesting drug candidate to be formulated in a transdermal patch, trying to improve pravastatin bioavailability and release (7-9).

The use of microneedles as an enhancer is a painless, minimally invasive, and easy to use manner. Microneedles increase the permeation of greater molecules than conventional chemical enhancers because the microneedles generate microchannels that increase the passage of them through the skin. The advantages that microneedles present have been demonstrated with previous studies of Prausnitz and Donelly (10, 11).

The purpose of this study was to know how the type of TP formulation and two different lengths of solid microneedles (MT Dermaroller™) affect the parameters of flux (F), time lag (t_Lag_), and permeability constant (Kp) on *in-vitro* percutaneous absorption studies.

## Experimental

Analytical grade reagents that comply with Analytical Chemistry Society (ACS) specifications were used: Pravastatin sodium (Moléculas finas), Chitosan (Sigma Aldrich), Glacial acetic acid (Meyer), Pluronic F-127 (PF-127) (BASF The Chemical Company), propylene glycol or PG (United States Pharmacopeia or USP), distilled water Milli-Q (Millipore Inc), 4-(2-hydroxyethyl)-1-piperazineethanesulfonic acid (HEPES) buffer (Sigma Aldrich), and MT Dermarollers™ approved by the FDA (lengths of 0.25 mm and 2.25 mm). 

*Transdermal patches preparation*

The transdermal patches (TP) were prepared by dissolving the appropriate amount of chitosan in acidified water, then the PF-127 and the rest of the ingredients were added to each formulation (Table 1). The films obtained were dried at room temperature for 72 h. The films were previously characterized and only the optimal formulations with the best mechanical characteristics were compared (12), they presented good characteristics like the acceptable uniformity of drug content, DSC studies showing no-interactions between excipients and drug, transdermal patches had good tensile strength, excellent bioadhesion and post wetting-bioadhesion, constriction was 0%, the thickness was less than 0.72 mm and kinetic release was adjusted to zero order kinetic model. These formulations were used since they allow to form the patches with suitable properties.

*In-vitro percutaneous absorption studies*

*In-vitro* skin permeation studies by passive diffusion were performed on the human abdominal skin of patients undergoing abdominoplasty. The Hospital Angeles Inn Chapultepec donated skin samples. The fatty and connective tissues were removed, and the samples were stored in a freezer at -21 °C for no more than 15 days. *In-vitro,* percutaneous absorption studies were performed using vertical Franz type diffusion cells under sink conditions. As a membrane between the two compartments, the abdominal human skin was used. The TP was placed on the skin. Receiver compartment was filled with a buffer solution of HEPES at pH 7.4. The assembly of the cells was placed on a magnetic stirrer with temperature control. The receiver solution was stirred with a magnetic bar and a thermostated at 32 °C. Sampling was performed at different intervals for 32 h and the drug content was determined by UV-Vis spectrophotometry at 238 nm the method was previously validated, and it complied with the specifications for linearity parameters (coefficient of determination r^2 ^˃ 0.99, slope coefficient CV ˂ 2%), accuracy and repeatability (coefficient of variation of replicates CV ˂ 2%). Cumulative drug accumulations per square centimeter of the formulations were graphed as a function of time (9, 12, 13). Three formulations of TP were evaluated (TP W, TP 1%, and TP 3%), two different lengths of microneedles were proved for TP formulations (0.25 mm, 2.25 mm) and passive diffusion studies for the TP were also performed. For the skin treated with microneedles, first the MT Dermarollers™ approved by the FDA (0.25 mm and 2.25 mm) was passed over the skin in different directions 10 times for each one (horizontal, vertical, and transverse). It was decided to use two microneedle lengths with a considerable difference in length (0.25mm y 2.25mm) with the intention of determining the effect of perforating the stratum corneum and the epidermis and reaching the dermis or not on the penetration of pravastatin through the skin.

## Results

The composition of transdermal patches and the results of the previous characterization of them by Serrano-Castañeda *et al*. 2015 are shown in Table 1 and Table 2. The statistical analysis used was ANOVA followed by a post hoc analysis (Tukey’s HSD). This analysis was done in compliance with the assumptions for ANOVA and post hoc analysis, giving a valid statistical analysis (14, 15).

*In-vitro percutaneous absorption studies*

**Figure 1.**
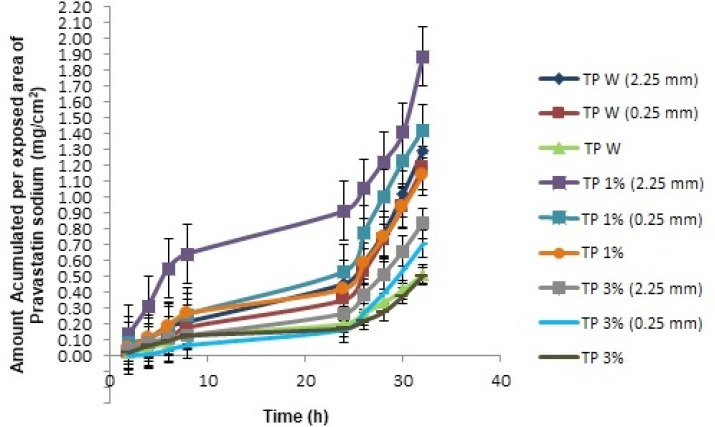
*In-vitro* Percutaneous absorption studies for each TP evaluated by passive diffusion with the corresponding length of microneedles used (0.25 mm, 2.25 mm and without using microneedles).

The kinetics of percutaneous drug absorption studies provides a good prediction of skin absorption *in- vivo.* In addition, these studies have low-cost, short test time, and reproducibility (16, 17). These studies calculated the cumulative amount (mg) of pravastatin and the amount accumulated per patch area. The latter was obtained by dividing the cumulative amount between 2.19 cm^2^ (area of ​skin exposed to the TP) and these values ​​were plotted to obtain the transdermal penetration profiles as a function of time (Figure 1). The parameters obtained are shown in Table (3).

**Figure 2:**
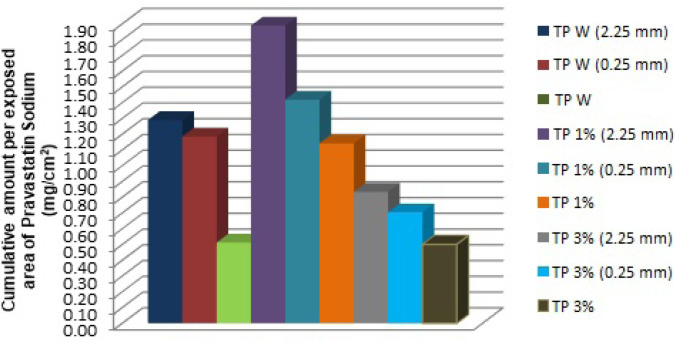
Comparison of cumulative amount per exposed area of pravastatin sodium (mg/cm^2^) of each TP with the two different lengths of microneedle used (0.25 and 2.25 mm and without using microneedles)**.**

The cumulative amount per exposed area (mg/cm^2^) of pravastatin sodium was graphed with the purpose of comparing all the formulations and different treatments to determine the highest value of an accumulated amount of the active ingredient through the skin (Figure 2)

Once the values were obtained, we proceeded to perform the statistical analysis with the software Statgraphics Centurion XV.II, to determine if there was a significant statistical difference between the formulations and the length of microneedles in the flux, time lag and permeability constant. A multifactorial ANOVA with Tukey’s HSD post* hoc* test was used for this purpose.

**Figure 3:**
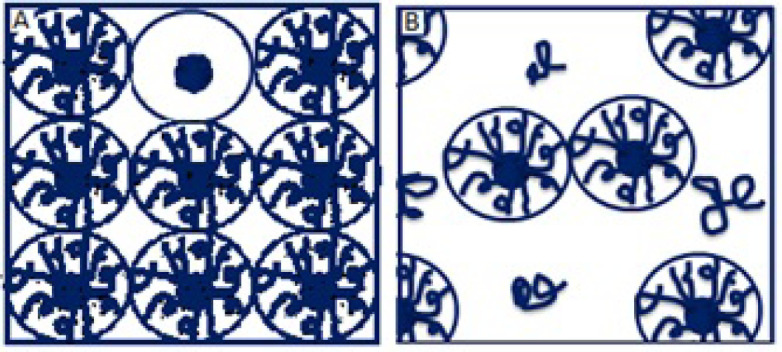
Schematic representation of PF-127 phenomena phases: A) shows the lattice or block phenomenon which prevents the release of the active and B) shows the micelle formation phenomenon

The accumulated quantities were analyzed with multivariate ANOVA, finding that the kind of TP 1% has an important effect on the kinetic parameters of *in-vitro* percutaneous absorption studies (*p* < 0.05). In Figure 1, we can see that the TP 1% coupled with microneedles of 2.25 mm, presented a greater amount of pravastatin sodium released compared to all transdermal patches, this is because PF -127 is an active non-ionic surfactant of amphiphilic character, which allows its polar portion (ethylene oxide chains) to have a greater interaction with the drug, increasing its release (18). The PF-127 also has the ability to be a structuring agent that forms a molecular framework depending on the concentration. By increasing its concentration, it gives rise to multimolecular aggregates (19) preventing the active principle from being released at a higher speed, since it is trapped inside this polymeric lattice; which explains the behavior of TP 3% and therefore a lower cumulative amount compared to the other transdermal patches (Figure 3).

However, the length of microneedles has an important role, because the microneedles of 2.25 mm, used in the three different formulations (TP W, TP 1%, and TP 3%), presented a greater penetration of pravastatin sodium. This is due to the thickness of the epidermis, the outermost layer of the skin where the stratum corneum is located, and the main permeability barrier varying from 0.04 to 1.6 mm of thickness (20); It is easy to traverse it using microneedles that allow the pravastatin sodium access to and through the skin, where it diffuses and is absorbed, thus improving drug administration. Therefore, having applied microneedles of different lengths, they can perform perforations at different depths and, to this extent, encourage the penetration of the active ingredient (21). This can be observed in the statistical analysis, so it was determined that the 2.25 and 0.25 mm microneedles show statistically significant differences (*p* < 0.05) with respect to the fact of not using microneedles in the TP.

Regarding the flow, this is defined as the quantity of the drug which crosses the membrane per unit area at a given time (mg / cm^2^*h) (22). Given the results obtained, it can be determined that for the type of TP and the length of microneedles used there is a statistically significant difference (*p* < 0.05), on the flux, based on the P-values obtained (TP type = 0.0106 and Microneedles = 0.0123).

In the case of microneedles, they increase the permeability of the skin because they break the stratum corneum. In the case of the type of TP, they present PF-127 and chitosan in the formulation that can form molecular frameworks depending on its concentration. Both polymers have the ability of forming polymer matrices. 

In our results we found that a statistically significant difference (*p* < 0.05) exists between TP 1% vs TP 3%. This behavior is given by the presence of PF-127 at the concentration of 1%, since in this concentration it allows the interaction with the drug, favoring a greater flow; while at 3% the formation of the molecular framework causes the drug to be trapped inside it, making it difficult for the pravastatin to be released at a higher speed (Figure 3), while the absence of PF-127 in the formulation does not present a significant difference (*p *> 0.05).

The permeability coefficient (Kp) allows us to determine the amount of the drug contained in the transdermal patch that passes through each centimeter of the membrane in a given time (cm/t) (23). The results obtained from the P-values (a type of TP = 0.0108 and microneedles = 0.0123) prove that the length of microneedles, as well as the type of TP present a statistically significant difference (*p* < 0.05).

This is due to the PF-127 that modulates the release of the drug and the formation of microchannels in the stratum corneum by the use of microneedles.

The lag time (t_Lag_) can be defined as the time of molecules saturating the membrane (23). According to the results, using different lengths of microneedles showed statistically significant lag time. It is established that the length of microneedles used has a statistically significant difference (*p* <0.05), determined by the P-value (Microneedles = 0.0379), while the types of patches do not present any statistically significant difference (*p *> 0.05) (P-values: TP type = 0.0670).

In the case of microneedles of 2.25 mm, they perforate the entire epidermis and part of the dermis, favoring the penetration of pravastatin sodium compared to the microneedles of 0.25 mm that only perforate the epidermis, and then there are barriers, preventing rapid absorption.

In the case of TP, these do not present a significant difference in all the formulations (TP W, TP 1%, and TP 3%). But the polymer matrix controls the release of the drug.

The determination of the surface area of the TP and the relationship with the absorption parameters were determined based on the Fick´s law. Therefore, according to the *in-vitro* percutaneous absorption studies, the possible sizes of the transdermal patches for the therapeutic dose equivalent to 10 mg Tablet are:

TP 1% with microneedles of 2.25 mm about ≈ 8 cm^2^, 

TP W with microneedles of 2.25 mm about ≈ 9 cm^2^, 

TP 3% with microneedles of 2.25 mm about ≈ 13 cm^2^, 

TP 1% with microneedles of 0.25 mm about ≈ 8.2 cm^2^, 

TP W with microneedles of 0.25 mm about ≈ 9 cm^2^ and finally 

for the TP 3% with microneedles of 0.25 mm about ≈ 13.5 cm^2^.

It should be mentioned that not only TP can release 10 mg of the drug per day, but also they can even release that dose or more for several days, modifying their area and the amount of drug with which they are loaded, and thereby avoiding the problems associated with the oral route. 

**Table 1:** Composition of transdermal patches

**Formulation**	**Chitosan (%w/w)**	**PG (%w/w)**	**PF-127 (%w/w)**
TP W	1.5	15	0
TP 1%	1.5	15	1
TP 3%	1.5	15	3
Acidified water	q.s		

**Table 2:** Results of previous characterization studies for each patch formulation

**Formulation**	**Drug content**	**Bioadhesion **	**Post wetting-bioadhesion **	**Tensile strength**	**Constriction **	**Thickness **	**Release**
	**(mg)**	**(g.f)**	** (g.f)**	**(g.f)**	**(%)**	**(mm)**	**Order 0 r** ^2^
TP W	19.16±1.86	60.148±18.533	27.913±9.061	184.80±91.210	0	0.41±0.01	0.9833
TP 1%	17.05±1.88	46.227±6.994	25.397±11.845	380.77±106.346	0.31	0.72±0.09	0.9912
TP 3%	21.18±1.99	51.049±24.036	42.010±9.210	192.14±178.794	0.31	0.71±0.02	0.9883

**Table 3:** Parameters of flux, time lag and permeability constant for each formulation with the corresponding length of microneedles (0.25 mm, 2.25 mm and without using microneedles) used for the *in-vitro* percutaneous penetration studies

**Formulation**	**Flux **	**Permeability constant X 10** ^-2 ^ **(cm/h)**	**Time lag (h)**
	(µg/cm^2^*h)		
TP W (2.25)	105.2	1.59	20.1644
TP W (0.25)	103.3	1.58	20.7609
TP W	38.5	0.59	18.9740
TP 1% (2.25)	115.2	1.75	16.7378
TP 1% (0.25)	111.4	1.70	19.1194
TP 1%	90.0	1.37	19.4833
TP 3% (2.25)	70.5	1.07	20.4454
TP 3% (0.25)	67.1	1.02	21.7973
TP 3%	40.9	0.62	20.4572

## Conclusion

The concentration of PF-127 only affects the flux and the permeability constant modulating the drug release from the matrix while the length of microneedles affects the three parameters (flux, permeability constant and lag time). This is because the microneedles are a transdermal penetration enhancer, which means that using them the process of absorption and diffusion of pravastatin sodium to and through the skin will be increased due to the microchannels generated by microneedles.

The TP 1% coupled with microneedles of 2.25 mm, is the one with the highest percutaneous absorption of pravastatin sodium through the skin. However, it is important to note that when using microneedles of 2.25 mm in each of the different formulations (TP W, TP 1%, and TP 3%), the amount of pravastatin sodium is increased in each case.
